# Three-Dimensional Printing Strategies for Enhanced Hydrogel Applications

**DOI:** 10.3390/gels10040220

**Published:** 2024-03-25

**Authors:** Hossein Omidian, Kwadwo Mfoafo

**Affiliations:** Barry and Judy Silverman College of Pharmacy, Nova Southeastern University, Fort Lauderdale, FL 33314, USA; km2796@mynsu.nova.edu

**Keywords:** 3D-printed hydrogels, tissue engineering, soft robotics, biofabrication, drug delivery systems

## Abstract

This study explores the dynamic field of 3D-printed hydrogels, emphasizing advancements and challenges in customization, fabrication, and functionalization for applications in biomedical engineering, soft robotics, and tissue engineering. It delves into the significance of tailored biomedical scaffolds for tissue regeneration, the enhancement in bioinks for realistic tissue replication, and the development of bioinspired actuators. Additionally, this paper addresses fabrication issues in soft robotics, aiming to mimic biological structures through high-resolution, multimaterial printing. In tissue engineering, it highlights efforts to create environments conducive to cell migration and functional tissue development. This research also extends to drug delivery systems, focusing on controlled release and biocompatibility, and examines the integration of hydrogels with electronic components for bioelectronic applications. The interdisciplinary nature of these efforts highlights a commitment to overcoming material limitations and optimizing fabrication techniques to realize the full potential of 3D-printed hydrogels in improving health and well-being.

## 1. Introduction

**Scheme 1:**
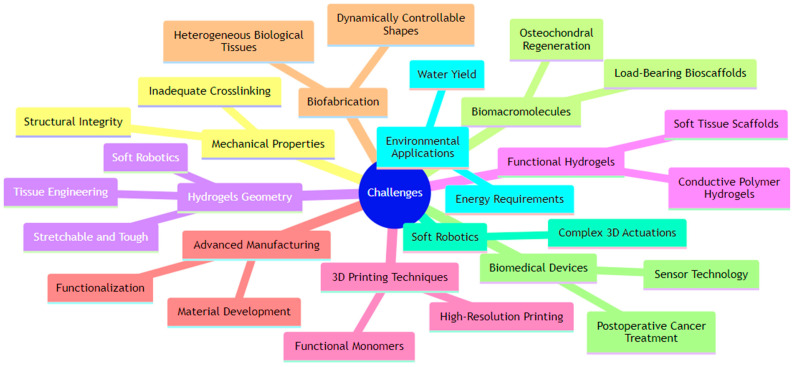
General challenges in 3D printing for biomedical applications.

The field of 3D-printed hydrogels has emerged as a dynamic and rapidly evolving area of research, driven by a diverse array of needs and challenges across various applications (Scheme 1). At the core of this innovative domain is the pursuit of advancing the customization, fabrication, and functionalization of hydrogels to meet specific requirements in biomedical engineering, soft robotics, tissue engineering, and beyond.

Customization of biomedical scaffolds for optimal tissue regeneration [1] remains a pivotal challenge, underscoring the importance of tailored solutions for individual patient needs. This necessity merges with the advancements in multimaterial 3D printing, enabling the creation of structures with unprecedented complexity and functionality. Researchers are also focused on enhancing the mechanical properties of bioinks [2], crucial for the realistic replication of biological tissues and the development of bioinspired actuators [3].

Fabrication challenges are pronounced in the domain of soft robotics, where the replication of soft material 3D printing [4] and embedding long fibers in hydrogels [5] are explored to mimic complex biological structures and functions. The quest for high-resolution, multimaterial, 3D hydrogel structures [6] further exemplifies the technical hurdles in achieving precise and scalable manufacturing techniques.

In tissue engineering, the creation of supportive environments for cell migration [7], nutrient/oxygen transport [8], and vasculature networks [9] is essential for the development of functional tissue constructs. This includes overcoming the natural limitations of hydrogel materials, such as the spread of wet droplets [10] and rapid solidification for photocrosslinking [11], to achieve desired geometries and properties.

The application of 3D-printed hydrogels extends into the domain of drug delivery [12,13], where controlled release mechanisms and biocompatibility are paramount. Similarly, the development of high-strength and ultratough hydrogels [14,15] addresses the need for materials that can withstand mechanical stresses while maintaining flexibility and biocompatibility for implantable devices.

Emerging applications also highlight the integration of hydrogels with electronic components [16,17], paving the way for innovative bioelectronics that can monitor and interact with biological systems in real-time. The development of conductive hydrogels [18,19] and self-healing materials [20,21] reflects the interdisciplinary nature of current research efforts, blending materials science with electrical engineering to create multifunctional devices.

The challenges and needs identified across these diverse applications demonstrate the interdisciplinary and innovative nature of research in 3D-printed hydrogels. From enhancing biocompatibility and mechanical strength [22,23] to achieving precision in nanoparticle arrangement [24] and optimizing scaffold fabrication [25], the field is characterized by a relentless pursuit of solutions that bridge the gap between theoretical potential and practical application. As researchers continue to address these challenges, the field of 3D-printed hydrogels stands on the border of significant breakthroughs that promise to transform a wide range of industries and improve human health and well-being.

## 2. Recent Studies on 3D-Printed Hydrogels

The study of 3D-printed hydrogels in the fields of material science and engineering is a rapidly evolving area focused on enhancing processing techniques, improving material properties, and integrating functional features. This work aims to expand the use of these materials across various disciplines. Key areas of research and development include the following:

1. Advancing photopolymerization methods and creating both thermoresponsive and biodegradable hydrogels to enhance their mechanical and physical characteristics.
2. Adding functional additives and creating new composites to address the limitations of hydrogels, thus improving their utility in sophisticated manufacturing and design applications.
3. Designing materials that replicate biological systems for use in biofabrication and tissue engineering, which involves improving mechanical properties, responsiveness to stimuli, and functional complexity. This opens new possibilities in regenerative medicine, soft robotics, and other areas.
4. Developing materials for tissue engineering and scaffolding that include bioactive molecules and porous structures, which support cell activity and closely resemble the natural tissue environment for advanced tissue regeneration.
5. Innovating in the field of biomedical devices and drug delivery by integrating bioactive molecules, enhancing mechanical and functional properties, and devising controlled release mechanisms tailored to specific medical treatments.
6. Advancing sensor technology and electronics with the use of conductive, responsive, and bioactive materials to create devices that are flexible, sensitive, and multifunctional. These devices have applications in wearable technology, soft robotics, and bioelectronics.
7. Focusing on the creation of advanced actuators and robotic systems for soft robotics, which are made from materials that exhibit superior mechanical properties and responsiveness for complex movements and mimicking biological functions.
8. Investigating the use of hydrogels in environmental and energy sectors to support sustainable energy production, agricultural development, and environmental clean-up, making use of their unique characteristics for innovative and sustainable solutions.

These initiatives demonstrate a strategic approach to leveraging 3D-printed hydrogels, emphasizing the continuous effort to refine material properties, broaden the scope of applications, and tackle significant challenges in science and technology.

## 3. Materials, Technologies, and Process Optimization of Three-Dimensional Printing

### 3.1. Hydrogel Crosslinking

Hydrogel crosslinking plays a pivotal role in hydrogel bioprinting, serving as the cornerstone for designing scaffolds that mimic the extracellular matrix, enabling the controlled release of bioactive molecules, and encapsulating living cells. Crosslinking methods, both chemical and physical, are integral for tailoring hydrogel properties such as degradation rates, mechanical strength, and biocompatibility, which are crucial for various biomedical applications.

Chemical crosslinking is highlighted for its versatility in creating hydrogels with robust mechanical stability. This method allows for the precise design of hydrogels that can degrade under physiological conditions into non-toxic products, ensuring biocompatibility. However, the use of potentially toxic crosslinking agents necessitates their complete removal to avoid adverse reactions with bioactive substances present within the hydrogel matrix [26]. In contrast, physical crosslinking methods offer an alternative that circumvents the use of toxic chemicals. These methods involve interactions that do not require covalent bond formation, thereby avoiding potential negative effects on the bioactive components embedded within the hydrogels. This advantage makes physically crosslinked hydrogels attractive for applications requiring high biocompatibility and functionality [26].

The development of interpenetrating networks through double crosslinking (covalent followed by ionic) not only enhances the hydrogel features but also allows for the modulation of hydrogel properties through the adjustment of preparation parameters. The use of neural network models to simulate and optimize these parameters further highlights the complexity and potential of hydrogel crosslinking in creating tailored matrices for drug delivery and cell encapsulation [27].

Exploring multifunctional crosslinking molecules has opened new avenues for regulating the degradation rates and mechanical properties of hydrogels. By comparing hydrogels formed with multifunctional versus bi-functional crosslinking molecules, it is evident that the former can provide superior mechanical stiffness and slower degradation rates, attributed to the increased crosslinking density and interaction points within the hydrogel network. This method demonstrates the importance of crosslinking chemistry in enhancing the performance of hydrogels for various biomedical applications [28].

Enzyme-catalyzed crosslinking represents an innovative and cell-friendly approach to hydrogel formation, offering a mild alternative to traditional chemical and physical crosslinking methods. This strategy enables the formation of covalently crosslinked hydrogels with complex architectures, mimicking the natural extracellular matrix more closely and providing dynamic scaffolds for tissue engineering and regenerative medicine. The shift towards enzymatic crosslinking features the evolving landscape of hydrogel preparation, where biocompatibility and functionality are paramount [29].

In situ crosslinking strategies for the 3D bioprinting of photo-crosslinkable hydrogels further illustrate the advancements in hydrogel technology. This approach enables the creation of complex, cell-laden constructs with high embedded cell viability and tunable cell behavior, featuring the critical role of crosslinking in the development of next-generation biofabricated tissues and organs [30].

Overall, the evolution of hydrogel crosslinking techniques—from chemical and physical to innovative enzymatic and in situ methods—demonstrates the field’s continuous advancement towards creating more sophisticated, biocompatible, and functional hydrogel systems for a myriad of biomedical applications.

### 3.2. Three-Dimensional and 4D Bioprinting

The transition from three-dimensional (3D) printing to four-dimensional (4D) printing represents a significant advancement in additive manufacturing, shifting from static constructs to dynamic, responsive structures. This evolution is characterized by the development of platforms that can change their properties, shape, or functionality over time in response to external stimuli, leveraging the capabilities of stimuli-responsive materials [31].

Three-dimensional printing has laid the groundwork for this advancement by enabling the precise layer-by-layer construction of complex structures, where control over the dimensions and properties of the printed objects is paramount. Among the materials employed in 3D printing, hydrogels have emerged as particularly promising due to their biocompatibility and the ability to undergo reversible transformations. These properties have made hydrogels a key material in the progression towards 4D printing, where the goal is to create structures capable of adapting to their environment [32].

The core of 4D printing technology lies in the use of stimuli-responsive hydrogels. These materials can expand, contract, or otherwise change shape in response to environmental triggers, such as temperature changes or the presence of specific chemicals. This responsiveness to stimuli has opened new avenues for creating devices and structures with applications ranging from actuators and cellular scaffolds to controlled drug release systems. The versatility and low cost of manufacturing these hydrogel-based structures further enhance their appeal for a wide range of applications [31].

An innovative approach in the field involves the development of composite inks, such as those combining cellulose with hydrogels. These composites not only maintain the responsive characteristics of hydrogels but also incorporate the mechanical strength of cellulose fibers. This combination enables the fabrication of structures that can morph according to predetermined designs in response to environmental stimuli, such as moisture levels. Such advancements illustrate the potential of 4D printing in creating more complex, responsive, and durable structures [33].

Among the specific applications of 4D bioprinting, the development of smart valves stands out. These valves, fabricated from a blend of alginate and poly(N-isopropylacrylamide), exemplify the practical application of 4D printing in producing structures that are not only mechanically robust but also capable of actuating in response to temperature changes. This highlights the potential of 4D-printed hydrogels in creating dynamic, responsive systems with possible uses in fluid management and soft robotics [34].

A notable technique in this evolution involves printing stimuli-responsive hydrogel structures with internal gaps using a combination of responsive and non-responsive pre-gel solutions. By printing these materials in a supportive medium and then solidifying them with ultraviolet light, it is possible to create complex structures capable of dramatic transformations in response to thermal stimuli. This method highlights the sophisticated control over material properties and structural design that 4D printing offers, paving the way for the fabrication of intricate, responsive patterns and shapes [35].

### 3.3. Hydrogel Bioprinting Techniques

Bioprinting is an emerging field that merges engineering, biology, and medicine to innovate in areas such as tissue engineering and regenerative medicine. This field is notable for its application of several key techniques, extrusion-based bioprinting, inkjet bioprinting, and vat photopolymerization, each with unique mechanisms and uses [36].

**Figure 1:**
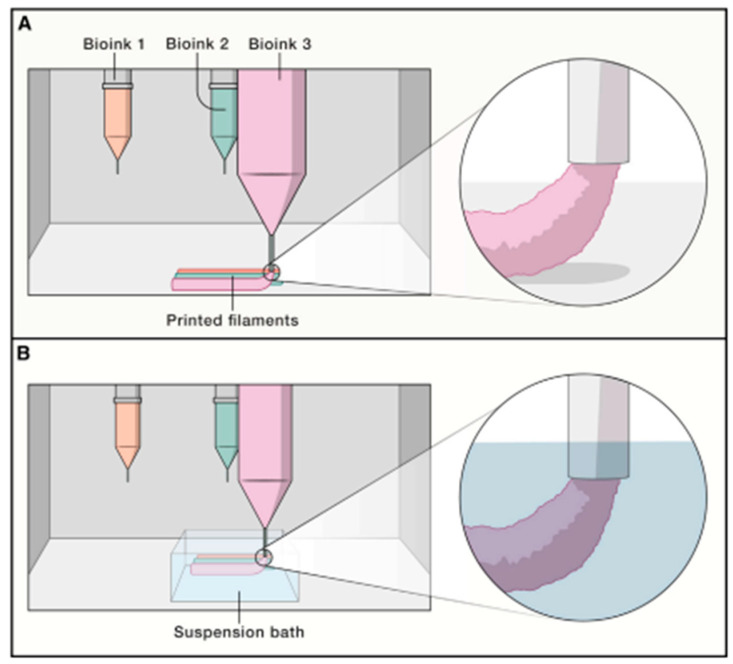
Extrusion printing (**A**) on a surface and (**B**) within a suspension bath [37].

Extrusion-based bioprinting (EBB) is akin to 3D printing, where biomaterials mixed with cells are pushed through a nozzle to build three-dimensional structures layer by layer. This method supports a broad viscosity range, accommodating various hydrogels and cell-laden bioinks, making it adaptable for creating large, intricate structures while preserving high cell viability. Despite its versatility, it struggles with lower resolution and potential shear stress that may harm cell viability and functionality. The performance of EBB is affected by factors such as the viscosity of the hydrogel, the diameter of the nozzle, and the speed of printing. Recent progress has expanded its applications to include printing tissues, organ modules, and microfluidic devices. Nevertheless, challenges persist, including limitations in organ fabrication, feature resolution, and regulatory hurdles, indicating a need for advanced bioprinting solutions and new bioink development to bridge the gap from laboratory to clinical use, aiming to produce viable products for tissue engineering and regenerative medicine [37,38]. Extrusion printing can be carried out on a surface or into a suspension bath or media. Figure 1 below shows how a bioink is deposited onto a surface or within a suspension bath with a pattern defined by the user.

Inkjet bioprinting utilizes thermal or piezoelectric actuators to place droplets of bioink on a substrate, noted for its high precision and resolution. This method is cost-effective and straightforward but is limited to bioinks with low viscosity and faces potential cell damage from heat or mechanical stress. Factors like bioink viscosity and minimizing cell damage are vital for its performance. The drop-on-demand jetting approach allows for the non-contact deposition of materials and cells, optimizing cell-to-matrix and cell-to-cell interactions. Despite its advantages, the effect of bioink properties on printing performance and cell health is not fully understood. Research indicates that bioink viscoelasticity and viscosity are crucial for printing accuracy and cell viability, emphasizing the importance of bioink formulation for precise cell deposition and the creation of adjustable cell spheroids [39,40].

**Figure 2:**
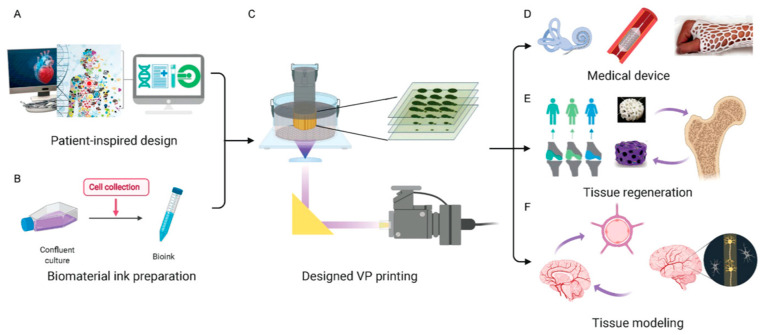
(**A**) Patient-specific designs from medical scanning images. (**B**) Biomaterial ink/bioink preparations. (**C**) The VP printing process in a layer-by-layer manner. (**D**–**F**) Exemplary applications of VP-printed constructs in biomedicine [42].

Vat photopolymerization involves the selective curing of photosensitive polymers or hydrogels with light to create solid 3D structures. It is distinguished by its high resolution and ability to produce complex shapes but is limited to photosensitive materials and the potential toxicity from photoinitiators. The technique’s performance depends on the choice and concentration of photoinitiators, as well as optimizing light intensity and exposure time. Recent developments in vat polymerization-based 3D printing and bioprinting have introduced new biomaterial ink formulations and system designs, with the expectation of their swift application in biomedical fields. This indicates a concerted effort to combine innovative vat polymerization techniques with biomaterial inks for better medical outcomes [41,42]. The patterns projected are typically derived from computerized 3D drawings or from CT scans or MRIs of patients’ resources. These patterns are designed using vat polymerization before remodeling to create patient-specific medical devices, functional human tissues for regeneration, and in vitro human-based tissue models for therapeutic screening, as illustrated in Figure 2 below.

In hydrogel bioprinting, selecting a bioprinting technique usually depends on the requirements of the desired application, such as the complexity of the structure, mechanical properties, and biological functionality. The composition of the hydrogel, the type of cells used, and the purpose of the application are crucial in determining the most appropriate bioprinting method. Ongoing research and development aim to address current limitations and expand the possibilities of bioprinting technologies, leading to the creation of more complex and functional bioprinted tissues and organs [43].

**Figure 3:**
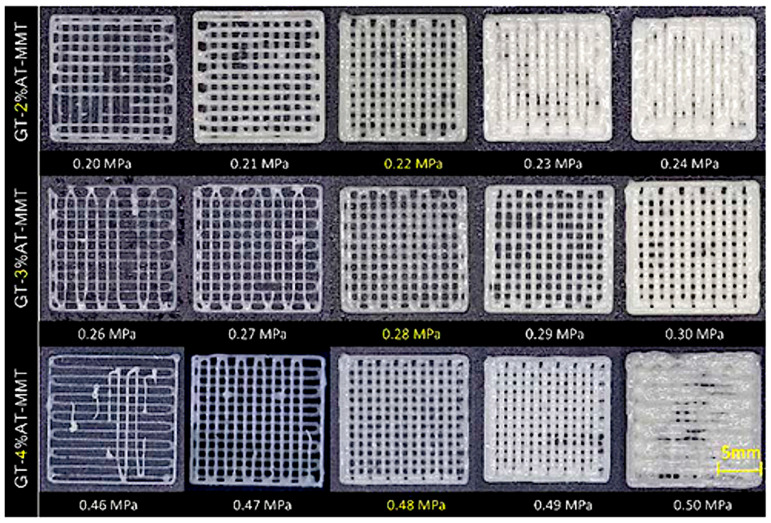
Sample morphologies printed with GT–AT–MMT bioinks of variable concentrations under predetermined pressures [2].

Recent advancements in 3D printing technologies, particularly in material and process optimization, have led to significant developments across various fields, including tissue engineering, regenerative medicine, soft robotics, and biomedical applications. These innovations demonstrate a concerted effort towards creating more sustainable, efficient, and customized solutions. Figure 3 below shows sample morphologies printed with GT–AT–MMT bioinks of variable concentrations under predetermined pressures.

In tissue engineering and regenerative medicine, the customization of bio-based scaffolds has been a notable achievement. These scaffolds, with controlled pore sizes and gradient structures, are designed to enhance tissue regeneration capabilities [1]. The optimization of 3D inkjet printing techniques for alginate bioinks has facilitated the creation of structures that closely mimic tissue, supporting physiological flows [10]. Additionally, the introduction of hydrogel-based technologies has marked a significant stride in cell therapy and tissue engineering applications, offering precise size control for cell therapy and tissue engineering applications [44] and optimizing cell-friendly fabrication for physiological models [9]. This is complemented by developments in bone regeneration, where MXene composite hydrogels have demonstrated synergistic antibacterial and osteogenic effects [45], and in drug delivery systems, where thermosensitive hydrogels show potential for controlled drug release [13].

The field of soft and marine robotics has also seen innovative applications of 3D printing technologies. The fabrication of jellyfish-mimic soft robot actuators [4] and biodegradable hydrogel actuators [46] illustrates the potential of these technologies in developing devices that operate effectively in challenging environments. Furthermore, the advancement in material properties, such as full-color luminescence and opacity tuning [3], enhances the adaptability and utility of robotic systems.

Hydrogel technologies have been central to overcoming fabrication limitations and introducing novel functionalities. The development of multiscale, multimaterial 3D hydrogel structures [6] and the improvement in mechanical properties through hydrogel composites [47] are exemplary. Moreover, photocrosslinking techniques for silk-fibroin-based hydrogels [11] and the use of a hydrogel pen for nanometer precision in 3D printing [48] highlight the ongoing efforts in process optimization.

Material science has played a crucial role in these advancements, with the exploration of hydrogel printability [49], rapid hydrogel bead fabrication [44], and the engineering of perfusable vasculature networks on-chip [9] being notable examples. These efforts not only enhance the practical applications of 3D printing technologies but also contribute to the broader understanding of material interactions and fabrication processes.

In conclusion, the advancements in 3D printing technologies stress the dynamic and interdisciplinary nature of this field. By pushing the boundaries of material and process optimization, these innovations offer promising solutions to complex challenges across science, medicine, and engineering. Table 1 below shows the different printing technologies, materials used and their applications.

## 4. Scaffold Mechanical Enhancement and Biocompatibility

**Figure 4:**
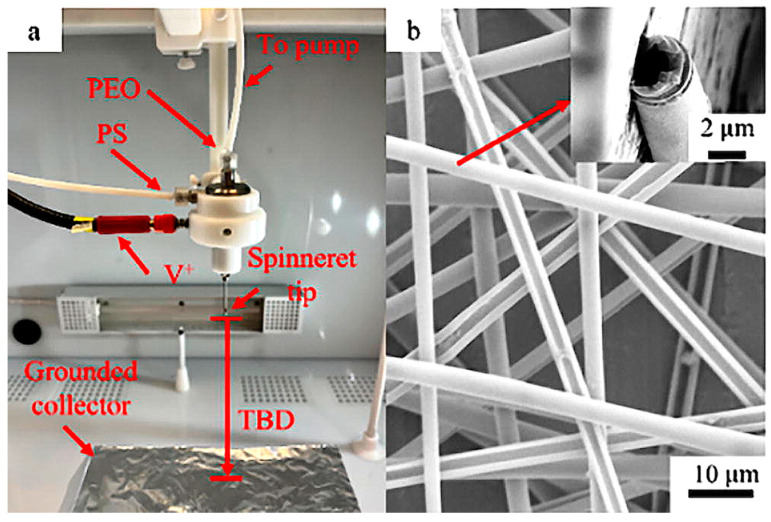
(**a**) Core–sheath spinneret; (**b**) SEM image of microtubules [23].

The recent advancements in scaffold design, particularly in enhancing mechanical properties and biocompatibility, signify a critical trend in tissue engineering, regenerative medicine, and material science. These advancements, as evidenced by numerous studies, are foundational to the development of scaffolds that support cellular growth and tissue integration effectively. For instance, the improvement in the mechanical properties of sodium alginate through the incorporation of agarose has been noted to facilitate cell growth, thanks to the scaffold’s designed micro-pores [56]. Additionally, the enhancement in hydrogel scaffolds with microtubes has shown promising results in terms of mechanical strength and biocompatibility, demonstrating the potential for application in soft tissue engineering [23]. Figure 4 below shows the core–sheath spinneret set up for the production of the microtubules and the SEM images of the microtubules produced.

The role of 3D printing technology in scaffold fabrication has evolved significantly, enabling the creation of complex structures tailored for specific applications. Innovations such as multiple-layered hydrogel scaffold printing [57], 3D-printed metal foams integrated with hydrogel for bone replacement [58], and the use of photopolymerizable copolypeptides for producing mechanically robust 3D objects [59] exemplify the versatility and precision offered by 3D printing techniques. These advancements highlight the industry’s move towards employing manufacturing technologies that allow for customized scaffold designs with enhanced functionality.

There is a growing emphasis on developing scaffolds that offer multifunctional capabilities, such as improved osseointegration, antibacterial properties [60], and conductivity conducive to neuron growth [61]. This trend towards multifunctionality reflects a shift in focus towards creating scaffolds that not only support tissue growth but also integrate additional features to enhance the overall therapeutic outcome.

Environmental sustainability and safety in scaffold materials have emerged as important considerations. The development of biodegradable cellulose hydrogel inks for 3D printing metal structures [62] represents a step towards more environmentally friendly and safer additive manufacturing processes. This focus on sustainability indicates a broader commitment within the field to reduce environmental impact and enhance patient safety through innovative material choices.

Moreover, the exploration of scaffold materials for applications beyond traditional tissue engineering showcases the adaptability and potential of these materials in cutting-edge fields. For example, the utilization of scaffolds in soft robotics [63], flexible electronics [16], and space agriculture [64] demonstrates the broad applicability and versatility of scaffold materials, paving the way for novel technological advancements.

In summary, the trends highlighted by recent research in scaffold mechanical enhancement and biocompatibility reflect a dynamic field characterized by innovation in material properties, manufacturing techniques, and application domains. The integration of enhanced mechanical and biological properties, coupled with the advancements in 3D printing technology, multifunctionality, environmental sustainability, and novel applications, emphasize the ongoing evolution of scaffold technology (Table 2).

## 5. Stretchable and Conductive Hydrogels

Recent advancements in the field of stretchable and conductive hydrogels have marked significant milestones across several applications, including but not limited to soft robotics, tissue engineering, human motion detection, and soft bioelectronics. These innovations are primarily driven by the integration of advanced printing techniques with hydrogel technology, the development of conductive hydrogels with enhanced functionalities, pioneering applications in soft bioelectronics and sensing, and innovations in sensing and energy generation.

The integration of advanced printing techniques with hydrogel technology has led to notable achievements. The high-resolution printing of stretchable double-network (DN) hydrogels has addressed the shaping challenges in soft robotics and tissue engineering, facilitated by quick lithography techniques [89]. Further, the field has seen the development of ultrastretchable and self-healing DN hydrogel printing, which exhibits remarkable mechanical strength and strain sensitivity, crucial for robotics and human motion detection [90]. Additionally, the introduction of a cryogenic 3D printing method for conductive hydrogel inks has produced cytocompatible, conductive scaffolds suitable for tissue engineering applications [91].

The development of conductive hydrogels with enhanced functionalities has also been a key area of progress. The coupling of 3D printing with interfacial polymerization has enabled the creation of conductive hydrogels with complex geometries, paving the way for the development of electroactive materials with precisely defined structures for advanced applications [92]. Moreover, the development of ultrasensitive and self-adhesive wearable devices with exceptional conductivity and stretchability has advanced motion monitoring and health detection, showcasing the potential of conductive hydrogels in wearable technology [92].

In soft bioelectronics and sensing, significant strides have been made. High-performance conducting polymer hydrogel-based Electrobiosignal Interfaces (EBIs) have improved ECG and EMG signal recording capabilities [93]. The fabrication of self-healable hydrogel–liquid metal composites for customizable multimodular sensor systems demonstrates practical advances in self-healing electronics [94]. Additionally, the reporting on 3D-printed implantable hydrogel bioelectronics for precise electrophysiological monitoring and electrical modulation emphasizes the potential in healthcare monitoring and therapies [95].

Furthermore, innovations in sensing and energy generation highlight the versatility of hydrogels. The rapid construction of double-network ionic conductive hydrogel for iontronic pressure sensors with biomimetic fingerprint microstructures enables tactile sensing and human motion detection [96]. The development of a 3D-printed conducting polymer hydrogel-based direct current (DC) generator for self-powered electromechanical sensing underlines the robust mechanical properties and flexibility, crucial for wearable electronics and environmental monitoring [97].

These advancements show the potential of conductive hydrogels to transform various industries by providing flexible, durable, and increasingly integrated solutions with electronic functionalities. The progress in this field is a testament to the collaborative efforts of researchers and engineers in pushing the boundaries of material science, electronics, and biomedical engineering (Table 3).

## 6. Self-Healing and Responsive Hydrogels

**Figure 5:**
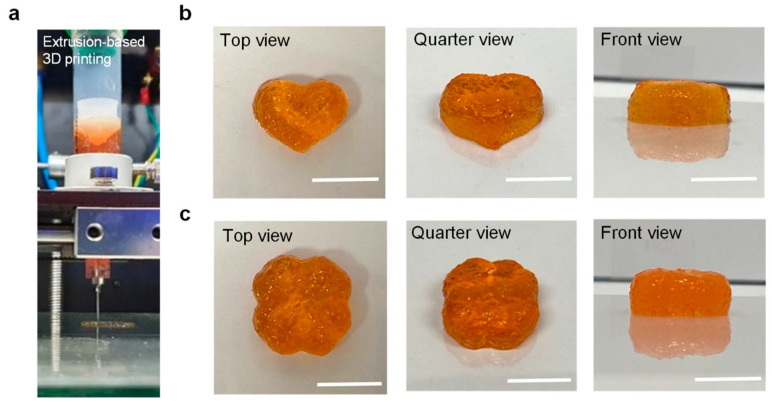
(**a**) Extrusion-based 3D printer, (**b**) different views of a heart-shaped 3D-printed structure, (**c**) different views of a clover-shaped 3D-printed structure [20].

The recent advancements in hydrogel technology, as evidenced by various studies, underscore significant progress in the fields of material science, 3D printing, and responsive actuators. A notable development includes the preparation of self-healing polyacrylamide (PAA)-based hydrogels tailored for extrusion-based 3D printing, which exhibit exceptional self-healing capabilities and rheological properties. This innovation enables the creation of diverse 3D structures that maintain integrity without deformation, enhancing the potential for practical applications [20]. Furthermore, improvements in the 3D printing resolution of Digital Light Processing (DLP) for high-water-content hydrogels have been achieved through the use of water-soluble photo-absorbers, which serve to enhance both mechanical properties and biocompatibility [101]. Figure 5 below shows a picture of an extrusion-based 3D printer and different views of 3D-printed structures.

The field has also seen the introduction of multi-responsive hydrogel actuators capable of anisotropic swelling, thereby enabling controllable motion. This development highlights the versatility of 3D printing in encoding complex behaviors within materials [102]. Similarly, photothermal responsiveness has been integrated into nanocomposite hydrogels, providing a pathway for actuators to exhibit controlled behaviors, thereby widening their application scope [103]. The incorporation of aligned graphene oxide (GO) within hydrogels has led to the realization of multi-stimuli-responsive actuation, facilitating complex architectural transformations [104].

On the front of innovative applications, there have been significant strides in developing hydrogel-based systems for sensing and soft robotics. A skin-like sensor incorporating a thermo-responsive hydrogel demonstrates temperature and pressure sensitivity, marking an advancement in wearable device applications and artificial intelligence [105]. Additionally, bio-hygromorphs that combine fish swim bladder hydrogels with 3D-printed scaffolds exhibit moisture-triggered morphing, presenting new possibilities for actuators and soft robotics [106]. The development of photothermal responsive hydrogels capable of ultra-fast and reversible deformation under near-infrared light exposure suggests potential uses in ultra-fast microrobotics for object manipulation [107].

The integration of hydrogels with 3D printing and electrospinning techniques has led to the creation of actuators with rapid response and enhanced designability in three-dimensional shapes [108]. This approach, along with the development of anisotropic models for hydrogel–fiber composites, enables the design of shape-morphing structures with tailored swelling behaviors [109]. The advancements in achieving the complex and controllable shape deformation of hydrogel architectures broaden their applications in soft robotics and tissue engineering [110].

In conclusion, the research and developments in hydrogel technology have led to a broad spectrum of applications ranging from 3D printing, soft robotics, to wearable sensors. These advancements, characterized by innovations in self-healing materials, multi-responsive actuators, and programmable shape transformations, highlight the material’s versatility and potential to address various challenges across multiple disciplines (Table 4).

## 7. Osteochondral and Bone Tissue Engineering

**Figure 6:**
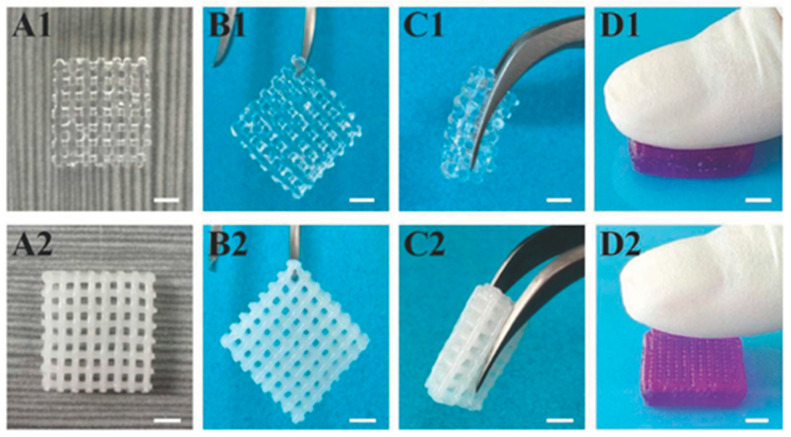
Macroscopic appearance of the printed porous (**A1**) PNT-35%-6 hydrogel scaffolds, (**A2**) PNT-35%-6-β-TCP-22% hydrogel scaffolds. Mechanical testing of the hydrogel scaffolds by (**B1**,**B2**) ability to support their weight, (**C1**,**C2**) twisting, and (**D1**,**D2**) compression [119].

Recent advancements in the field of osteochondral and bone tissue engineering have been marked by significant progress in scaffold development, emphasizing the balance between mechanical strength, biodegradability, and biological performance. A pivotal aspect of this progress is the development of high-strength, biohybrid gradient hydrogel scaffolds, which exhibit controllable architecture and mechanical properties, making them suitable for direct 3D printing applications [119]. This is complemented by the fabrication of biodegradable supramolecular-polymer-reinforced-gelatin hydrogel scaffolds tailored for osteochondral regeneration, highlighting an emphasis on both enhanced mechanical strength and biological performance [120]. Such advancements emphasize the industry’s focus on creating supportive environments conducive to tissue repair and regeneration. Figure 6 below shows mechanical testing of pristine PNT-35%-6 hydrogel scaffolds and its TCP biohybrid.

In hydrogel scaffolds, notable innovations include the development of beta-sheet-reinforced NP hydrogels, which boast improved mechanical properties and anti-swelling capabilities, beneficial for osteochondral tissue regeneration. These scaffolds facilitate cell support and differentiation, showcasing the potential of bilayer structures in tissue engineering [121]. Additionally, hybrid hydrogel scaffolds have been developed for bone tissue engineering, demonstrating improved osteogenesis and cell adhesion capabilities. This highlights the potential of 3D printing technology in creating scaffolds that support complex tissue engineering applications [122].

The application of 3D printing and bioprinting technologies represents a significant trend in tissue engineering, offering innovative solutions for fabricating scaffolds with precise structural and mechanical properties. For instance, the creation of gelatin/HA/3D-graphene scaffolds with enhanced mechanical properties signals a move towards scaffolds that can better mimic the physical and biological environment of native tissues [123]. Furthermore, the adoption of 3D bioprinting for the development of tissue-engineered heart valves demonstrates the versatility of these technologies in producing viable constructs for in vitro testing and potential clinical applications [124].

Novel scaffold designs have been introduced to promote enhanced tissue regeneration, incorporating drug-release mechanisms and bioactive materials to stimulate specific biological responses. Alg/Gel ink containing MBG and ZIF-8 has been used to develop bone scaffolds with drug-release capabilities, aiming to enhance bone regeneration (e.g., [125]). Hybrid scaffolds have also been shown to improve osteogenic and angiogenic performance, offering promising solutions for the regeneration of large bone defects (e.g., [126]).

Furthermore, efforts to improve tissue integration and angiogenesis have yielded significant results, particularly in the context of wound healing and bone regeneration. For example, 3D-printed alginate/gelatin-based scaffolds have demonstrated enhanced tissue integration and angiogenesis, crucial for diabetic wound healing [127]. An ECM-enriched hydrogel in a 3D-printed scaffold has shown potential in enhancing vascularized bone formation, emphasizing the importance of scaffold design in supporting not only cell growth but also integration with the surrounding tissues and promoting blood vessel formation [128].

In conclusion, the trends in osteochondral and bone tissue engineering reflect a comprehensive approach towards developing scaffolds that support effective tissue regeneration. These developments illustrate a commitment to overcoming the challenges inherent in tissue engineering, aiming to offer innovative solutions for tissue repair and regeneration (Table 5).

## 8. Drug Delivery Systems and Therapeutic Applications

The exploration of innovative drug delivery systems and their therapeutic applications has seen significant advancements, focusing on improving treatment efficacy, minimizing side-effects, and enhancing patient compliance through the utilization of novel materials and techniques. Three exemplary developments in this field include fabricated hydrogel scaffolds for cancer therapy, hydrogel contact lenses for the treatment of diabetic retinopathy, and DNA-induced biomineralization techniques for 3D-printed wound dressings.

Fabricated hydrogel scaffolds have been developed for targeted cancer therapy, demonstrating a capacity for controlled drug release directly at the tumor site, which significantly reduces systemic toxicity associated with conventional chemotherapy treatments. This approach not only offers a more personalized and targeted therapy but also aims to enhance the efficacy of postoperative treatment, aligning with the trend towards more patient-specific cancer treatments. The precise control over drug release exemplified by these hydrogel scaffolds represents a significant step forward in the field of oncology, aiming to improve therapeutic outcomes while reducing adverse effects [138].

In ophthalmology, hydrogel contact lenses have been explored as a novel drug delivery system for diabetic retinopathy, a leading cause of blindness globally. This development marks a shift towards non-invasive, accessible treatments, offering a simpler and cost-effective alternative to traditional invasive procedures. The use of drug-eluting contact lenses points to a broader trend in healthcare towards improving treatment accessibility and patient compliance, facilitating easier management of chronic conditions such as diabetic retinopathy [139].

Furthermore, the introduction of a DNA-induced biomineralization technique for the creation of 3D-printed wound dressings highlights a significant innovation in wound care, particularly for diabetic wounds. This technique enhances the biological activity of dressings, promoting accelerated healing and reducing infection risks. By leveraging DNA sequences to induce biomineralization, this approach illustrates the convergence of biotechnology and material science, aiming to produce wound care solutions that are not only effective but also biologically active, setting a new standard in the treatment of complex wounds [140].

These developments collectively reflect a broader trend towards leveraging advanced technologies and materials to address specific healthcare challenges, emphasizing the importance of personalized treatment, minimal side-effects, and enhanced patient compliance (Table 6).

## 9. Hydrogel Testing and Evaluation

The exploration of 3D-printed hydrogels involves a comprehensive range of testing methods and evaluations, aiming to optimize their utility for varied applications. This analysis highlights the importance of customizing hydrogel properties to meet the specific requirements of diverse fields such as biomedical applications, mechanical enhancements, and responsive functionalities. Through a detailed review, several key trends in testing methodologies emerge, showcasing the interdisciplinary nature of hydrogel research and development.

A pivotal area of focus is on the mechanical properties and structural integrity of hydrogels, with a significant body of work [2,22,23,49,56,57,59,60,62,65,71,72,73,74,80] emphasizing the evaluation of tensile strength, compressive strength, elasticity, toughness, and viscoelastic properties. This emphasis is crucial for applications where mechanical integrity is paramount, such as tissue engineering scaffolds [119,121,122,129] and soft robotics [63]. Various methods, including traditional mechanical testing, rheological measurements [47], and optimization of sintering processes [62], highlight the diverse strategies employed to enhance and assess hydrogel mechanical performance. Ultrasound-based techniques such as macro-indentation [141], multimode ultrasound viscoelastography (MUVE) [142], and dual-mode ultrasound elastography (DUE) [143] provide precise, non-invasive methods to measure the mechanical properties of hydrogels. These methods enable the estimation of elastic moduli, and the characterization of viscoelastic properties with high spatial and temporal resolution. MUVE excels in measuring viscoelastic behavior in soft hydrogels, offering superior sensitivity and resolution compared to traditional methods [142]. DUE allows for the non-invasive creep testing of hydrogel biomaterials, providing insights into the material’s stiffness and viscoelastic properties [143]. Additionally, ultrasound elastography has potential applications in medical diagnostics, such as differentiating between normal uterine tissue and pathological conditions [144]. These ultrasound techniques are instrumental in advancing research in mechanobiology and tissue engineering by accurately characterizing the microscale mechanical properties of biomaterials.

Another trend is the focus on biocompatibility and cellular interactions, with numerous studies [9,13,44,45,54,55,58,83,84,85] investigating cell viability, proliferation, differentiation, and specific biological responses. These assessments are vital for hydrogels intended for regenerative medicine, drug delivery systems, and tissue engineering, where the material’s compatibility with biological systems is a key determinant of success. Techniques such as confocal microscopy, cell patterning, and co-culture perfusion [9,10,54] play a crucial role in evaluating the hydrogels’ biomedical efficacy.

There is also a concerted effort to optimize the 3D printing process and integrate functional capabilities within hydrogels, as seen in studies [1,4,6,10,11,50,145]. This research highlights the importance of refining printing techniques to achieve gradient structures, embedded functionalities, and precise architectural control, catering to advanced applications such as microvasculature fabrication [10] and embedded microchannels [6].

The development of responsive or “smart” hydrogels [102,103,104,107,108,109,110,111,112,115,118] represents a forward-thinking trend. These materials are designed to alter their properties in response to external stimuli, targeting applications in actuation, sensing, and controlled drug release. This move towards dynamic, adaptable materials signifies a shift towards hydrogels capable of performing complex, responsive tasks.

Furthermore, the research on stretchable and conductive hydrogels [17,89,90,92,93,94,95,97,100] is gaining traction. Evaluations of electrical conductivity, stretchability, self-healing abilities, and gauge factors are critical for applications in wearable electronics, sensors, and bioelectronic interfaces, highlighting the potential of hydrogels that merge mechanical durability with electrical functionality.

In conclusion, the array of testing methodologies applied to 3D-printed hydrogels features the material’s adaptability and the broad spectrum of potential applications. From structural and mechanical assessments to biocompatibility evaluations and the integration of functional features, the current trends in testing reflect a concerted, interdisciplinary effort to tailor hydrogel properties to specific needs. As the research in this field continues to evolve, refining these testing methods will play a crucial role in unveiling new applications and enhancing the capabilities of hydrogel-based systems.

## 10. Limitations of Using 3D Printed Hydrogels

In the domain of 3D printing technology, materials, and process optimization, hydrogels have faced difficulties in achieving the requisite mechanical properties for specific biomedical applications, such as load-bearing tissue regeneration. This is attributed to their lack of strength and durability [2,5]. Efforts to enhance biocompatibility and biofunctionality are ongoing, focusing on the incorporation of cell-signaling molecules and the creation of bioinspired scaffolds [45,54]. Despite innovative approaches, such as the development of multi-hydrogel vasculature systems, vascularization remains a critical challenge for the survival and integration of engineered tissues [9]. Furthermore, the field grapples with achieving high resolution and fidelity in 3D-printed structures [7,50], compounded by the complexity of regulatory landscapes for clinical applications [7,9,45,50,54].

Optimization efforts towards scaffold mechanical enhancement and biocompatibility have yet to fully succeed in matching the mechanical properties and elasticity of native tissues [23,56,57,58]. The pursuit of universal biocompatibility to promote desired cellular activities continues [14,67,68], alongside engineering scaffolds that support vascularization and integration with host tissues, particularly for complex constructs [58,66]. High-resolution printing and regulatory requirements pose significant barriers to clinical adoption [7,50,58,66].

The creation of stretchable and conductive hydrogels, which maintain mechanical flexibility and electrical conductivity under stress, presents its own set of challenges [89,90,92]. Further optimization is necessary for their integration with biological tissues for applications in bioelectronics and tissue engineering [91,93]. Issues of scalability, reproducibility, self-healing efficiency, degradation rates, and environmental impact remain significant hurdles [21,94,98,99,100].

For self-healing and responsive hydrogels, ensuring long-term durability under repetitive stress conditions is a challenge [20,102,103]. Developing hydrogels that respond rapidly to stimuli requires further optimization [101,104,111]. Scalability and the simplification of manufacturing processes, without compromising functionality, are essential for sustainable development, alongside integration with electronic and biomedical systems and addressing environmental stability and degradability [105,106,107,110,112,113,115,117,118].

In osteochondral and bone tissue engineering, attaining the necessary mechanical strength for load-bearing applications remains a challenge [119,121]. Essential factors include long-term biocompatibility, bioactivity, adequate vascularization, and controlled degradation rates for effective regeneration and integration [122,123,125,126,127,128,129,130,131]. Scalability and the reproducibility of fabrication also pose manufacturing challenges [124,133,134].

Finally, in drug delivery systems and therapeutic applications, developing precise and controllable drug release profiles is elusive, necessitating tailored kinetics [138,139]. Ensuring long-term biocompatibility, minimizing potential toxicity, and achieving predictable degradation rates are critical for efficacy [138,140]. Moreover, the scalability of production and enhancing patient compliance, especially for hydrogel-based devices like contact lenses, are vital for commercial viability and therapeutic effectiveness [139,140]. This comprehensive overview highlights the multifaceted challenges in the development of 3D-printed hydrogels, underlining the need for interdisciplinary approaches to overcome these obstacles and advance the field.

## 11. Future Directions

The future of 3D-printed hydrogels is poised at the confluence of innovation and optimization, spanning materials, processes, and technologies.

### 11.1. Three-Dimensional Printing Technology, Material, and Process Optimization

The evolution of material innovation is fundamental, emphasizing the development of new hydrogel formulations to precisely emulate target tissues. This includes the integration of smart materials capable of responding to environmental stimuli [55] and fostering cellular activities [13], alongside hybrid approaches that combine Direct Ink Writing with melt electrowriting [47] to improve both resolution and mechanical properties. Computational modeling and design are also highlighted as key for achieving more accurate scaffold designs, leveraging predictive modeling within CAD/CAM tools to align closely with physiological requirements. Furthermore, research focusing on in vivo integration and functionality, such as immune response, degradation, and tissue remodeling [45,54], is deemed critical for the clinical success of printed structures. The development of clear regulatory guidelines and standards is identified as essential for smoothing the regulatory approval pathway.

### 11.2. Scaffold Mechanical Enhancement and Biocompatibility

The field is advancing through novel crosslinking methods [15,18,19] and the incorporation of functional materials [69,70,71], aiming to strike a balance between strength, biocompatibility, and biodegradability. Enhanced bioprinting techniques [74,75,76] and the integration of bioactive molecules [60,72,73] are paving the way for more intricate and functional tissue constructs. The development of smart and responsive hydrogels [77,78,79] heralds new prospects for medical devices and drug delivery systems. Furthermore, the emphasis on customization and patient-specific solutions emphasizes the importance of interdisciplinary collaboration and personalized medicine [84,85,146].

### 11.3. Stretchable and Conductive Hydrogels

Innovations are merging hydrogels with materials like liquid metals [94,100] and conducting polymers [93,95], aiming to surpass current limitations and augment functionality. The development of next-generation implantable devices is being driven by advanced manufacturing techniques [21,89,91] and biohybrid interfaces [93]. Additionally, the exploration of smart sensing systems for health monitoring and environmental sensing [96,97] emphasizes the quest for sustainable and biodegradable solutions [99].

### 11.4. Self-Healing and Responsive Hydrogels

The field is moving towards emulating complex biological processes through self-healing hydrogels [102,103,115] and precision 3D printing techniques [101,110,111]. This is complemented by material development inspired by bioinspired design principles [108,113,118], with a focus on sustainable production [106,107].

### 11.5. Osteochondral and Bone Tissue Engineering

The enhancement in tissue regeneration mimicry is being addressed through advanced material combinations and hybrid scaffolds [25,119,121,123,129,137], along with smart hydrogels [102,103,135]. Achieving control over scaffold architecture [110,111,122] is essential, as is the conducting of in vivo and clinical studies [126,128,131].

### 11.6. Drug Delivery Systems and Therapeutic Applications

The advancement of targeted therapy is being pursued with smart and responsive hydrogels capable of controlled drug release, integrating nanotechnology [138,140] and advanced fabrication techniques [139,140]. Addressing clinical, regulatory, sustainability, and ethical aspects is crucial for the responsible development of these systems [138,139,140].

In conclusion, the progression of 3D-printed hydrogels represents a holistic approach that blends material innovation, fabrication advancements, in vivo functionality, regulatory compliance, and interdisciplinary collaboration. Such concerted efforts are vital for addressing existing challenges and unlocking the extensive potential of hydrogels in various applications.

## 12. Conclusions

The advancements in 3D-printed hydrogels have revolutionized various fields, offering solutions to complex challenges in tissue engineering, drug delivery, soft robotics, and beyond. By customizing hydrogels, tissue engineering has seen significant progress, with bio-based scaffolds improving tissue regeneration and microvasculature engineering enhancing functionality. Bioprinting technologies have become more versatile, enabling complex structures and sacrificial techniques for enhanced nutrient transport. In soft robotics, hydrogels have enabled the creation of responsive actuators and environmentally sustainable systems. Drug delivery systems benefit from controlled release mechanisms and personalized treatment options. Despite challenges like mechanical strength and regulatory hurdles, ongoing research aims to optimize materials and processes, ensuring the continued evolution and widespread adoption of 3D-printed hydrogels across diverse applications.

## Figures and Tables

**Table 1:** Three-dimensional printing technologies, material, and process optimization.

Printing Technology	Material and Printable Concentrations	Printing Parameters and Resolution	Cell Viability (If Applicable) and Application	Ref. #
3D printing	Sodium alginate, gelatin, and cellulose nanocrystals; glutaraldehyde and calcium chloride as crosslinkers	Optimized for uniform and gradient pore structures, pore sizes of 80–2125 µm	Biomedical scaffolds for tissue repair and regeneration	[1]
3D bioprinting	Gelatin–alginate–montmorillonite (GT-AT-MMT)	Optimized with variable AT content	Tissue engineering, drug delivery, regeneration medicine	[2]
3D printing (Direct Ink Writing)	Lanthanide-ion-coordinated supramolecular hydrogel	Multimaterial direct ink writing, controlled shape, luminescence, and opacity	Soft actuators with tunable luminescence and opacity	[3]
3D printing	Hydrogel soft actuator	Air pressure-controlled contraction, mimics jellyfish contraction ratio	Jellyfish-mimic soft robots	[4]
Continuous Fiber Extruder (CFE) for FRESH	Long fibers embedded in hydrogels	Compatible with desktop 3D printers	Long-fiber embedded hydrogel 3D printing for enhanced mechanical properties	[5]
Hybrid Laser Printing (HLP)	PEGDA, GelMA hydrogels	Sequential additive and subtractive modes, microscale resolution	Multiscale, multimaterial 3D structures for optics, photonics, biomedical sciences	[6]
Two-Photon 3D printing	hydrogel for “hydrogel-in-hydrogel”	Precisely controlled channel-like hydrogel confinements, high-resolution hydrogel microstructures	Breast cancer cells migrate faster in stiffer confinements, studying cancer cell migration in confined 3D environments	[7]
3D printing with sacrificial templates	GelMA, PVA for sacrificial templates	PVA templates dissolved to form channel networks, channels promote oxygen/nutrient supply and cell growth	Hydrogel constructs with channel networks for 3D cell culture, tissue repair	[8]
Two-Photon 3D printing and Scaffold Molding	Gelatin-based ink for printing, fibrin hydrogel	Optimized printing parameters and fibrin composition, perfusable multi-hydrogel vasculature	Supports growth and proliferation of human lung fibroblasts and endothelial cells, engineering perfusable vasculature models on-chip for tissue models	[9]
3D Inkjet Printing	Alginate	Optimized reaction at the single-droplet level, tissue-like microvasculature	Live cells can be patterned, structuring hydrogels into tissue-like geometries	[10]
3D printing with Photoinduced Self-crosslinking	Silk fibroin, tyramine-modified CMC-Na, XG, Ru(bpy)3Cl2, KPS	Extrusion-based printing with post photocrosslinking, ~550 µm × 1000 µm pores	Silk-fibroin-based composite hydrogel scaffolds for tissue engineering	[11]
3D-printed PCL-Blended Scaffold	PCL, PVA(-)PAA hydrogel, sodium indomethacin	Evaluation of mechanical, thermal, and porosimetric properties	Load-bearing tissue damage support with anti-inflammatory drug delivery	[12]
3D printing	CNCs, PCLA copolymers, DOX, lysozyme	In situ-forming thermosensitive, injectable, 3D-printable, stable 3D structures up to 10 layers	Controlled biodegradation, in vivo antitumor effect, controlled release, breast cancer treatment	[13]
3D printing	Fmoc-FF hydrogel, silver or gold nanoparticles	Sensitive and reproducible SERS templates, low-concentration detection (as low as 100 pM)	SERS-based sensing	[24]
3D printing	Low-melting-point agarose, sodium alginate	Solution flow rate (250–500 µL/min), stir speed (500–1250 rpm), bead diameters: 5–150 µm depending on conditions	MC3T3-E1 cells can survive and proliferate, preparation of hydrogel beads for cell therapy and tissue engineering	[44]
3D printing	GelMA/beta-TCP/sodium alginate (Sr(2+))/MXene	MXene composite for photothermal antibacterial and osteogenic abilities	Biocompatibility and bone formation ability shown, bone regeneration in infected bone defects	[45]
FRESH 3D printing	Calcium-alginate hydrogels from brown seaweed	Thin-wall structures that are water-tight and pressurizable, small-scale actuators	Biodegradable, safely edible by marine organisms, biodegradable hydrogel actuators for marine robotics	[46]
Melt electrowriting (MEW)	Poly(ε-caprolactone), polyacrylamide, pHEMA hydrogels	Sinusoidal fiber deposition, radial-architecture for stabilizing fibers, diameter = 13.3 ± 0.3 µm, height = 330 µm	Improving shear properties of hydrogel–fiber composites	[47]
3D printing	Electrolyte-filled hydrogel pen	Localized electrochemical reaction, small sizes (110 nm in diameter), tall heights (up to 30 µm)	Electrochemical applications, 3D printing in 3 dimensions	[48]
Extrusion-based bioprinting	Alginate, gelatin, methyl cellulose	Flow behavior and mechanical properties analyzed, examined pore size, strand diameter	3D hydrogel scaffolds for bioprinting	[49]
Stereolithography	Hydrogel for double molding; PDMS	High-resolution stereolithographic prints, high-resolution features replicated in PDMS	Engineered tissue design, microheart muscles	[50]
3D-printing-enabled microfluidics	GelMA, perfluorodecalin-oil-coated	Varying GelMA concentrations (7–15% *w*/*v*) for different sizes (35–250 µm diameters), cell-laden hydrogel microspheres	High long-term viability for human osteosarcoma Saos-2 cells, cell-laden hydrogel microspheres for tissue engineering	[51]
3D printing by solvent exchange	N-heptyl-D-galactonamide in DMSO/water	Continuous extrusion	Biocompatible structures, temporary supports or sacrificial ink	[52]
3D-printed protein hydrogel cages	Protein hydrogel	pH-stimulated reversible shape changes, microscale device dimensions	Shaping giant membrane vesicles for studying cell division and polarity	[53]
3D printing	dECM, MSC-derived exosomes	Tissue-specific bioinspired scaffolds	Promotes MSC attachment, spread, migration, proliferation, differentiation, cartilage, and subchondral bone regeneration	[54]
3D printing	UV-responsive CSMA, CaCO_3_, porous titanium	UV-responsive chitosan hydrogel mineralized with CO_2_ diffusion	Promoted adhesion and proliferation of BMSCs, cell proliferation and osteogenesis in vitro	[55]

**Table 2:** Three-dimensional hydrogel compositions for scaffold mechanical enhancement and biocompatibility.

Printing Technology	Material and Printable Concentrations	Printing Parameters and Resolution	Cell Viability (If Applicable) and Application	Ref. #
3D printing	Poly(vinyl alcohol) (PVA) and chitosan (CS) with dual-physical crosslinking	Cyclic freezing–thawing followed by sodium citrate solution soaking, tensile strength of 12.71 MPa, Young’s modulus of 14.01 MPa	High-performance hydrogels for engineering, intelligent machines, and soft robotics	[14]
3D printing	Hydrogel with titin-like domains	Multiple nozzles for topological design, enhanced extensibility, and toughness, mimicking titin toughening principle	Ultratough hydrogel structures for materials/structures with desired properties	[15]
Extrusion printing	Polyacrylamide hydrogel and poly(dimethylsiloxane) (PDMS)	Lithium-chloride-containing hydrogel printed onto treated PDMS, scaling resistance under tension and fatigue	Transparent and conductive integrated device fabrication	[16]
Digital Light Processing 3D printing	Poly(3,4-ethylenedioxythiophene) polystyrene sulfonate (PEDOT:PSS) and poly(2-hydroxyethyl acrylate) hydrogel–elastomer hybrids	Submillimeter-scale device resolution, high conductivity and superior electrical stability	Conductive hydrogel–elastomer hybrids for stretchable electronics	[17]
Photo-curing 3D printing	PHEA-PSS hydrogels with in situ-polymerized 3,4-ethylenedioxythiophene (EDOT)	Double network structure for complex spatial structures, tensile strength close to 8 MPa, electrical conductivity of 1.2 S cm^−1^)	High-strength conductive polymer hydrogels for flexible sensors	[18]
Vat photopolymerization	UV-curable hydrogel with polymerizable micelles, glycerol, and plant cellulose	Rapid layer-by-layer printing, 6 s per layer, enhanced mechanical toughness, 4.55 MJ/m^3^	Strain sensing for flexible electronic devices	[19]
Fused Filament Fabrication (FFF)	PLA surfaces with atmospheric-pressure plasma jet (APPJ) treated and UV-grafted composite hydrogel (HEMA, PEGMA, HAp)	APPJ treatment and UV photo-grafting	Improved biocompatibility with osteoblast-like MG63 cells, bone scaffolds	[22]
3D printing	Carboxymethyl cellulose/sodium alginate hydrogel with polystyrene microtubes or poly(ethylene oxide)	Viscosity, filament size, swelling, tensile, and compressive tests	Soft tissue scaffolds	[23]
Direct Ink Writing	Sodium alginate and agarose composite hydrogel	Submicron pores	Tissue engineering scaffolds with enhanced mechanical properties and conducive for cell growth	[56]
3D printing	Hydroxybutyl chitosan (HBC) affected by ion effects	Temperature and salt concentration control	Cytocompatible, cartilage tissue repair with elaborate structure and good mechanical properties	[57]
Electron Beam Melting (EBM)	Ti-6Al-4V foam with hydrogel matrix containing deferoxamine mesylate (DFM)	60% porosity, tensile strength of 18 GPa	Pre-osteoblasts and HUVECs show increased survival and proliferation, biomedical bone replacement implants with vascularization	[58]
Direct Ink Writing (DIW) / Direct Laser Writing (DLW)	Photopolymerizable copolypeptides (hydroxy ethyl-L-glutamine, vinylbenzyl-L-cysteine)	Polymer topology and/or molecular weight control, tailored for high-resolution macroscale or microscale 3D structures	Multipurpose hydrogel resins for 3D printing with adjustable mechanical properties	[59]
3D Printing	PEEK implants with surface modifications of drug-loaded photocured hydrogel (acrylic acid-acrylamide with simvastatin-cefepime)	Surface sulfonation and hydrogel modification, improved hydrophilicity, reduced surface roughness	Enhanced biocompatibility and osseointegration capacity, orthopedic and dental implants	[60]
Direct Ink Writing	Poly(2-hydroxyethyl methacrylate) (pHEMA)/pyrrole ink	Printing through nozzles as small as 1 μm, good biocompatibility, ~94.5% viability with Aplysia californica neurons	High cell viability, conductive hydrogel platforms for neuron growth and electrophysiological recording	[61]
Materials extrusion	Biodegradable cellulose hydrogel inks with metallic powders (316 L stainless steel, copper)	Sintering at 1050 °C for 316 L and 985 °C for Cu, 31.92–32.07% porosity	Biomedical and energy applications with optimized porous metallic structures	[62]
DLP micro-3D printing	Electroactive hydrogel microstructures	Digital light processing technique for precise dimensional control, large deformation response	Soft robotic manipulation and locomotion	[63]
3D printing	Carboxymethyl cellulose hydrogel for soybean sprouting substrate	Printed into different dimensions for soybean sprouting, influence of bean orientation on sprouting process	In-space seed nursery with hydrogel-based seed planter	[64]
3D printing	Swollen chitosan microspheres in various functional monomer inks	Acid-driven tunable swelling ratio, strength (0.4–1.01 MPa), dissipated energy (0.11–3.25 MJ/m^3^), elongation at break (47–626%)	Soft device production including 4D printing robots and wearable strain sensors	[65]
3D printing	Porous hydrogel scaffolds with medium pore sizes (100–1000 μm)	Medium pore sizes (100–1000 μm)	High activity and osteogenic differentiation of BMSCs, promotes viability and osteogenic differentiation of BMSCs in hydrogel scaffolds	[66]
3D printing	Dialdehyde cellulose nanocrystals (DAC) and gelatin (GEL)	Optimal printing conditions including pressure and nozzle speed, additional crosslinking time, and temperature	Confirmed biocompatibility, tissue repair scaffolds	[67]
3D printing	Cellulose nanocrystal (CNC), hard phenyl acrylate (PA), soft acrylamide (AAm) for interpenetrated polymer network	UV irradiation for crosslinking, tensile strength up to 16.5 MPa, toughness up to 6 MJ m^−3^)	Artificial meniscus with equivalent or superior mechanical performance to human meniscus	[68]
Photopolymerization	Zeolite/polymer-based hydrogel composite, FAU-13X zeolite (~40 wt%)	Visible LED light irradiation at room temperature, elastic modulus increased by more than 100% with 10% FAU-13X	Water absorption materials and 3D printing applications	[69]
Extrusion 3D printing	Cellulose solution in dimethyl sulfoxide (DMSO) and tetrabutylammonium hydroxide (TBAH)	Controlled extrusion and solvent-exchange with deionized water, compression strength of 96 kPa	Preparation of natural cellulose hydrogel scaffolds	[70]
3D printing	Nanocellulose/polyethyleneglycol diacrylate (PEGDA) hydrogel	Light curing, 40 s of light curing for 1.8% nanocellulose mixture, maximum compression modulus of 0.91 MPa	Rapid prototyping of nanocellulose structures with controlled architecture	[71]
Direct Ink Writing (DIW) 3D Printing	Photocurable hydrogel with hydroxyethyl acrylate, gelatin, crosslinker, and photoinitiator	Cured with 405 nm visible light, adjustable mechanical properties from soft and fragile to rigid and tough	Printing of 3D objects with adjustable mechanical properties	[72]
3D printing	Poly(3-sulfopropyl acrylate, potassium salt) (PSPA) with glycidyl methacrylated hyaluronic acid (GMHA) as a crosslinker	Stretchability increased to 49%, fracture toughness to 40 J/m^2^	Electroactive hydrogels for soft robotics	[73]
Extrusion-based gradient printing	Tough alginate/poly(acrylamide) hydrogel and acrylated urethane-based UV curable adhesive material	Custom software to control dispensing rates for material gradients, continuous gradient of modulus	Bioinspired structures like artificial tendons	[74]
Embedded 3D printing	Various hydrogel inks in supportive baths (gelatin slurry, agarose fluid gel, Carbopol, oil-based baths)	Supportive bath matching for shapeability, dominated by diffusion-driven or charge-driven mechanisms	Tissue engineering to soft robotics	[75]
3D printing and hydrogel integration	Soft polymeric materials for structure and different hydrogels for active components	Integration of hydrogel into 3D-printed constructs	Plant-inspired actuators	[76]
3D Printing	Hydrogel with carbon nanotubes (PEGDA/MWCNT) for swelling-behavior-based detection	Swelling behavior for liquid detection, effective identification of liquid leakage position and volume	Flexible liquid sensors for biomedical engineering and environmental science	[77]
3D printing	Poly(N-isopropyl acrylamide) with clay and phytic acid crosslinked polyaniline	Rheological properties modified for printing, high porosity, electrical conductivity, thermal response sensitivity	Thermo-responsive electroconductive hydrogel for motion sensors	[78]
3D printing	Alginate-methylcellulose hydrogel with magnetic nanoparticles (MNPs)	Rheological properties influenced by MNPs, good mechanical stability	Magnetic hydrogel actuators for soft electronics, robotics, biomedical devices	[79]
3D printing	High-strength supramolecular polymer hydrogel with poly(e-caprolactone) (PCL) and poly(N-acryloyl glycinamide) (PNAGA)	Radial and circumferential orientation to imitate collagen fibers, Young’s moduli of 20.15 MPa circumferentially and 10.43 MPa radially	Implanted in rabbit knee joints for 12 weeks, meniscus substitute with anisotropic microarchitecture	[80]
Microstereolithography	PEGDA photopolymerized hydrogels	Influence of curing parameters and ink formulation on structure and swelling, graded profile of curing and submicron pores	Impact on hydrogel structure and swelling behavior	[81]
Extrusion-Based 3D printing	DEAE-cellulose, alginate, gelatin hydrogel composites	Varying concentrations for optimal properties, improved shear thinning, viscosity, and mechanical integrity	Excellent hemocompatibility and cytocompatibility, tissue engineering biomaterial inks	[82]
Extrusion-based 3D printing	Chitosan methacrylate (CHMA) and polyvinyl alcohol (PVA) hybrid hydrogel microparticles	Self-healable pre-crosslinked hydrogel microparticles for high fidelity and biocompatibility, self-supportive yield strength (540 Pa) for printed construct fidelity	Supports growth of bone-marrow-derived mesenchymal stem cells, biomimetic scaffolds for tissue engineering	[83]
3D printing	Chitosan methacrylate with various drugs for burn treatment	Customizable wound dressings via 3D printing	Biodegradable and biocompatible, personalized hydrogel wound dressings for thermal burns	[84]
3D printing	Supramolecular polymer hydrogel with poly(e-caprolactone) and poly(N-acryloyl glycinamide)	Radial and circumferential orientation for structural integrity, Young’s moduli of 20.15 MPa circumferentially	Meniscus substitute with biomimetic structure	[85]
3D printing	Thermo-responsive p(NIPAm-co-PEGDA) hydrogel with iron oxide nanoparticles	Additive fabrication of anisotropic hybrid 3D structure, superior light absorption properties	Direct solar vapor generation (SVG) device	[86]
3D printing	SnS2/SnS nanocomposite hydrogel	Utilized as a photoenhanced triboelectric nanogenerator (PTNG), enhanced performance under light illumination	Energy harvesting and self-powered devices	[87]
3D printing	Composite hydrogel with alginate and rice husk biochar	Designed for organic contaminants adsorption in tap water, significant adsorption capacities for IBU and MB	Water and wastewater treatment for organic contaminant removal	[88]

**Table 3:** Stretchable and conductive 3D-printed hydrogel compositions.

Printing Technology	Material and Printable Concentrations	Printing Parameters and Resolution	Cell Viability (If Applicable) and Application	Ref. #
Suspension 3D printing	Liquid metal in self-healing hydrogel	Gel concentration, nozzle inner diameter, flow rate, printing speed, customizable droplet size	3D macrostructures and stereo-electronic systems of liquid metal for flexible electronics	[21]
Optical Projection Lithography	DN hydrogels (covalent and ionic)	Hydrogel composition, crosslinker and photoinitiator concentrations, laser light intensity, high resolution	Soft robotics, tissue engineering	[89]
3D printing	Kappa-carrageenan/PAAm DN hydrogel	UV exposure	Demonstrated excellent recoverability and self-healing capability, strain sensors for robotics and human motion detection	[90]
Cryogenic 3D printing	Chitosan (CS) and edge-functionalised expanded graphene (EFXG)	Custom cryogenic extrusion, control over ink and surface temperatures, as small as 200 µm	Supports NSC-34 mouse motor neuron-like cell viability, attachment, and proliferation, engineering of 3D-structured excitable cells	[91]
3D printing/Interfacial Polymerization	Poly(ethylene glycol)diacrylate, pyrrole to polypyrrole (PPY)	Digital light processing system, complex and defined geometry	Conductive hydrogel creation	[92]
3D printing	PEDOT:PSS, PVA, ionic macromolecular dopants	Composite engineering, freeze–thawing for EBI, high resolution	EBI-based skin electrodes for ECG and EMG signal recording	[93]
3D-printed Stamp	Hydrogel–liquid metal composite	Molds for electrical pathway, liquid metal filling, customizable	Self-healable electrodes for multimodular sensor systems, biosignal detection	[94]
Direct Ink Writing (DIW)	PEDOT:PSS	Superior 3D printability, tissue-like mechanical compliance, instant tough bioadhesion	Implantable bioelectronics for electrophysiological monitoring and modulation	[95]
Digital Light Processing 3D printing	Acrylamide/acrylic acid, magnesium chloride (DN hydrogel)	Biomimetic fingerprint MH film, mechanical property tunability, 150 µm	Iontronic pressure sensor for tactile sensing and human motion detection	[96]
Continuous 3D printing	Conductive hydrogel with TPMS architecture	Self-powered DC energy generation upon mechanical stimulation, high flexibility with over 50% compressive strain	Self-powered electromechanical sensing for wearables, robotics, AI, environmental monitoring	[97]
3D printing	Acrylamide and alginate DN hydrogel, MOF ligands	Prepolymer mixing, shear-thinning agent, copper ions for in situ MOF growth	Wearables, implantable and flexible sensors, chemical separations, soft robotics	[98]
Projection microstereolithography (PμSL)	Conductive crosslinked hydrogel	Temperature, pH, and chemical composition adjustments, ultra-fast programmable	Degradable, conductive wearable devices, human–machine interface	[99]
In Situ 3D printing	Liquid metal (LM)–hydrogel hybrids	Direct fabrication with mechanical compliance to soft tissues	Multifunctional soft bioelectronics and devices	[100]

**Table 4:** Self-healing and responsive 3D-printed hydrogel compositions.

Printing Technology	Material and Printable Concentrations	Printing Parameters and Resolution	Cell Viability (If Applicable) and Application	Ref. #
Extrusion-based 3D printing	Poly(acrylic acid)-based hydrogel with 1.0 mol% covalent crosslinker and 2.0 mol% dynamic crosslinker	G’~1075 Pa and tan delta~0.12	Self-healing hydrogel for 3D shape fabrication	[20]
Digital Light Processing (DLP) 3D printing	High-water-content hydrogel with water-soluble photo-absorber nanoparticles	Water-soluble photo-absorbers for high resolution, lateral resolution: 5 µm, vertical resolution: 20 µm	High-resolution, high-water-content hydrogel structures	[101]
Stereolithography 3D printing	Multi-responsive hydrogel-based actuators with functionally graded structures	Layers with different volume expansion properties, crosslinking density, chemical composition, high resolution	Hydrogel actuators with anisotropic swelling for rapid, controllable motion	[102]
3D printing via ultraviolet light polymerization	Poly(N-isopropyl acrylamide)/graphene oxide (PNIPAm/GO) nanocomposite hydrogel	Optimal shear thinning properties adjusted by nanoclay, graphene oxide as infrared light absorber	Photothermally responsive actuators and functional programming	[103]
Direct-Ink-Writing Printing process	Graphene oxide (GO) flakes in sodium alginate (SA) matrix	Spontaneous alignment of GO flakes by shear force, locally controlled orientation	Multi-stimuli-responsive actuation with site-specific anisotropy	[104]
3D printing	Thermo-responsive hydrogel	Capacitor circuit incorporation	Skin-like sensors for AI, wearable devices, human/machine interaction	[105]
Fused Deposition Modeling (FDM) 3D printing	Fish swim bladder hydrogel and wood flour-filled polylactic acid (WPLA)	Bio-hygromorph construction	Environment actuators, meteorosensitive architectures	[106]
Projection microstereolithography 3D printing	N-isopropylacrylamide-based photothermal responsive hydrogel	Grayscale variation for adjustable deformation, rapid prototyping of complex 3D structures	Ultra-fast photothermal responsive shape memory hydrogel for microrobots	[107]
Combining 3D printing with electrospinning	Stimuli-responsive hydrogels on electrospun membranes	Patterns printed for morphing behaviors guided by swelling/shrinkage mismatch	Rapid deformed hydrogel actuators with enhanced designability	[108]
Direct Ink Writing (DIW)	Hydrogel–fiber composites	Controlled fiber distribution by printing direction and nozzle size	Soft active materials, shape-morphing structures	[109]
Stereolithography-based 3D printing	Single-material hydrogel with secondary microstructures	Programmable microstructures for asymmetrical swelling	Soft robotics, tissue engineering, actuators	[110]
Additive manufacturing	Poly(electrolyte) complex, BZ reactions	Varied shapes, sizes, angles for spatiotemporal patterns	Smart structures, biological oscillators	[111]
Various 3D printing techniques	Stimuli-responsive hydrogels (SRHs)	Focus on printing techniques, stimuli mechanisms, shape-morphing behaviors	Tissue engineering, drug delivery, soft robots, sensors	[112]
3D-printed Hydrogel–elastomer systems	Soft elastomer and hydrogel for anisotropic swelling	Star-like to circular geometry mimicking cactus	Soft robotics, controlled movements mimicking natural organisms	[113]
Hierarchical Filament 3D printing	Poly(ethylene glycol) diacrylate, poly(acrylic acid) microgels	Laminar flow for crosslink density organization	Soft actuators, sensors with programmed shape transformations	[114]
Extrusion-based 3D printing	Magnetic hydrogel (PNIPAm embedding Fe3O4 nanoparticles) and elastomer composite	Shape transformation under alternating magnetic field	~50% cancer cell killing via magnetic hyperthermia during deformation, hyperthermia cancer therapy	[115]
Microreactive Inkjet Printing (MRIJP)	Alginate hydrogel	Controlled droplet collision and gelation dynamics, <40 cP viscosity for reactive inks	Tissue-mimicking structures, 3D microstructures	[116]
3D printing	Hydrogel with photoactive titanium dioxide nanoparticles, alginate, gelatin, and dyes	Optimization of viscosity and ink composition for mechanical properties	UV exposure sensors, wearable sensors	[117]
3D printing	Iron platinum (FePt) nanoparticle-embedded pentaerythritol triacrylate (PETA), poly N-isopropylacrylamide (pNIPAM), and poly N-isopropylacrylamide acrylic acid (pNIPAM-AAc)	Pollen-grain-inspired microrobot	Multifunctional microrobots for biomedical applications	[118]

**Table 5:** Three-dimensionally printed hydrogel compositions in osteochondral and bone tissue engineering.

Printing Technology	Material and Printable Concentrations	Printing Parameters and Resolution	Cell Viability (If Applicable) and Application	Ref. #
3D printing with Lyophilization	Photocurable hydrogel of gelatin and hyaluronic acid	Methacrylic anhydride and a photoinitiator used, followed by lyophilization	Scaffolds supported mature cartilage regeneration	[25]
Direct 3D printing	Thermoresponsive supramolecular copolymer hydrogel (N-acryloyl glycinamide and N-[tris(hydroxymethyl)methyl] acrylamide)	Shear thinning, immediate gelation on cooled substrate	Facilitates attachment, spreading, and chondrogenic and osteogenic differentiation of hBMSCs for repair of osteochondral defect	[119]
3D printing	PACG-GelMA (Poly(N-acryloyl 2-glycine) and methacrylated gelatin)	Photo-initiated polymerization	Supports cell attachment and spreading, enhances gene expression of chondrogenic and osteogenic differentiation for osteochondral Regeneration	[120]
DLP 3D printing	GelMA-SFMA (methacrylated gelatin and methacrylated silk fibroin) with ethanol treatment	Digital Light Processing (DLP)	Supports cell attachment and spreading, facilitates osteogenic differentiation for osteo-chondral tissue regeneration	[121]
Extrusion-based 3D printing	SF-SA (silk fibroin and sodium alginate) with 2.5% MgP (magnesium phosphate)	Extrusion-based 3D printing	Promoted adhesion of rat mesenchymal stem cells, improved osteogenesis for bone tissue engineering	[122]
Hydrogel 3D-printing	Gelatin/hydroxyapatite/3D-graphene	Hydrothermal autoclave, hydrogen gas injection		[123]
3D bioprinting	Hydrogel with encapsulated valve and mesenchymal stem cells	Photoencapsulation, cell-hydrogel printing	High cell viability for tissue-engineered heart valves	[124]
3D printing	Alginate/Gelatin hydrogel with Bioactive Glass 45S5 and ZIF-8 nanoparticles	Shear-thinning behavior for extrusion	Higher MG-63 cell viability (>90%) than Alg/Gel alone for bone tissue regeneration	[125]
3D printing with hydrogel integration	Alginate or alginate–bioglass composite hydrogels with 3D-printed poly(lactic acid)	Surface treated with polyacrylic acid (PAA)	Good cell viability and proliferation, promotes osteogenic differentiation and calcium mineralization for bone tissue engineering	[126]
3D printing	Alginate/gelatin-based hydrogel with CaCO_3_ microspheres	Pre-crosslinking with Ca^2+^ for increased printability, pore area: 4.43 +/− 0.14 μm², line diameter: 184 +/− 25 μm	Improved NIH-3T3 cell proliferation for diabetic wound healing	[127]
3D printing	ECM-enriched hydrogel in a PCL scaffold		Excellent viability and osteogenic activity of BM-MSCs for Vascularized Bone Regeneration	[128]
3D printing	Hydrogel with nanohydroxyapatite gradient (pure hydrogel, 40% nHA/60% hydrogel, 70% nHA/30% hydrogel)		Superior repair results with BMSC-loaded gradient scaffolds for repair of osteochondral defects	[129]
3D printing	Cellulase-laden cellulose nanofiber/chitosan hydrogel composites	Enzyme-mediated biodegradation	Excellent cytocompatibility (supports fibroblast cell attachment, proliferation, and growth) for tissue engineering	[130]
3D printing	Titanium with tri-calcium-phosphate-loaded demineralized bone matrix hydrogel		Biocompatible, promotes osteogenesis for bone regeneration	[131]
FDM and Photo-curable Hydrogel Printing	PLA microstructure with gelatin hydrogel and bioactive gold nanoparticles (GNPs)		Enhanced osteogenic differentiation of ADSCs for bone tissue regeneration	[132]
Direct vs. indirect 3D printing	Methacrylamide-functionalized gelatin	Direct extrusion and indirect printing with PLA mold	Supports adipose stem cell adhesion and differentiation for adipose tissue regeneration	[133]
3D printing with hydrogel coating	PCL/PU scaffolds with PDA-coated decellularized matrix hydrogel		Promotes cell survival, proliferation, and ECM production for temporomandibular joint disc repair	[134]
3D printing with magnetic hydrogel	Chitosan hydrogel with embedded iron oxide nanoparticles	Use of external magnetic force, nanoparticles modified with citrate	Increased osteoblast proliferation and differentiation for bone tissue regeneration	[135]
3D printing	Sodium alginate/hydroxyapatite hydrogel ink, pre-crosslinked	Further crosslinking post printing to enhance mechanical properties, macro-porous structure, >80% porosity	Enhanced mBMSC adhesion for bone tissue engineering	[136]
3D bioprinting	GelMA/nHAp and THA hydrogels	Optimal printing temperature of 20 °C	High cell viability in both GelMA/nHAp and THA hydrogels for osteochondral tissue modeling	[137]

**Table 6:** Three-dimensionally printed hydrogel compositions in drug delivery and therapeutics.

Printing Technology	Material and Printable Concentrations	Printing Parameters and Resolution	Cell Viability (If Applicable) and Application	Ref. #
3D printing	Pluronic F127 and sodium alginate, DOX, and copper ions (F127-SA/Cu-DOX hydrogel scaffold)		Postoperative chemo/chemodynamic therapy for cancer	[138]
3D printing	PHEMA hydrogel, Avastin-loaded	Simple, cost-effective design, comparable to commercial lens	Ocular drug delivery for diabetic retinopathy	[139]
3D printing	DNA from salmon sperm, DNA-induced biosilica, hydrogel inks	Optimized for effective exudate and blood absorption, mechanical tunability	Diabetic wound healing	[140]

## Data Availability

Not applicable.
